# Alan L. Hart: An Innovative Pioneer in Radiology and Transgender Rights

**DOI:** 10.7759/cureus.87485

**Published:** 2025-07-07

**Authors:** Athena Reich, Katharine A Colbert

**Affiliations:** 1 LGBTQ+ Health, Orlando College of Osteopathic Medicine, Winter Garden, USA; 2 School of Nursing, Boston College, Chestnut Hill, USA; 3 Public Health, University of New England, Biddeford, USA

**Keywords:** biographies, chest radiography, gender-affirming surgery, historical vignette, lgbtq+, medical innovation, public health, radiology, transgender, tuberculosis

## Abstract

Robert Allen Bamford Jr., also known as Alan L. Hart (1890-1962), was an American physician, radiologist, researcher, and novelist who developed and implemented the gold standard for tuberculosis (TB) screening using chest radiography, two decades before epidemiological tools became widespread. His groundbreaking medical contributions were made despite facing systemic discrimination as one of the first transgender men in the United States to undergo gender-affirming surgery. This article explores Dr. Hart’s achievements in medicine and public health, his gender transition, and his lasting impact on both healthcare and lesbian, gay, bisexual, transgender, queer, intersex, asexual, and other non-binary or non-heterosexual identities (LGBTQIA)+ history.

## Introduction and background

**Figure 1:**
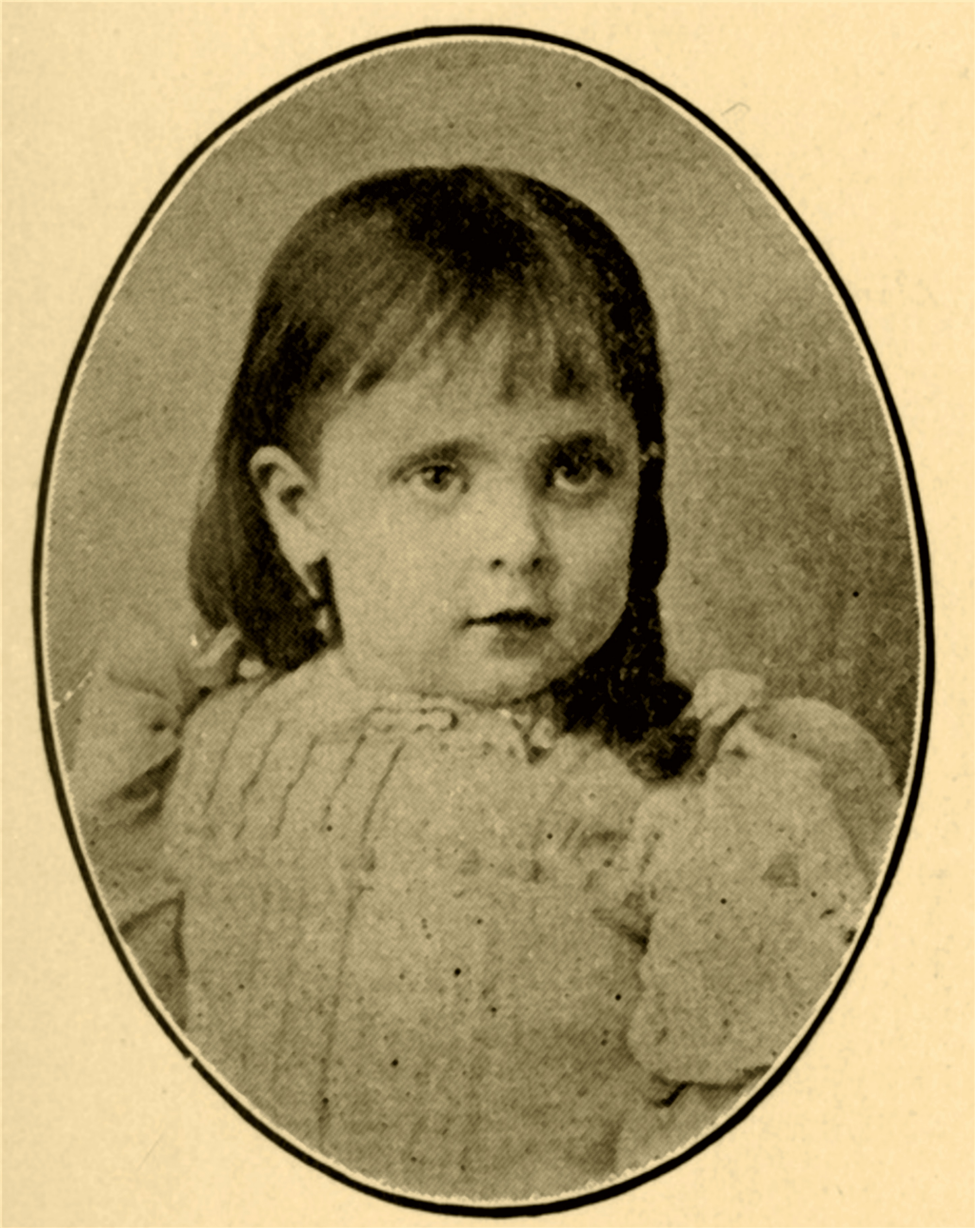
“Baby Lucille Hart” (1894) Used under a Creative Commons Attribution 4.0 International License (CC BY 4.0). Courtesy of Lewis & Clark College Special Collections and Archives, Dr. Alan L. Hart Collection, Portland, Oregon. Available from: https://specialcollections.lclark.edu/items/show/91 Rights information: https://creativecommons.org/licenses/by/4.0/

Alan L. Hart was born biologically female as Alberta Lucille Hart in Halls Summit, Kansas, in 1890 [1]. After his father died of typhoid fever, Hart’s mother relocated the family to Oregon, where he was raised. Hart exhibited a persistent male identity from early childhood, expressing a preference for boys’ clothing and toys made by his grandfather. While his mother reportedly dismissed his gender expression as “foolish”, his grandparents referred to him as their grandson in their obituaries - a remarkably affirming act, especially for that era [2]. As shown in Figure 1, Hart was known as Lucille in childhood before later transitioning.

**Figure 2:**
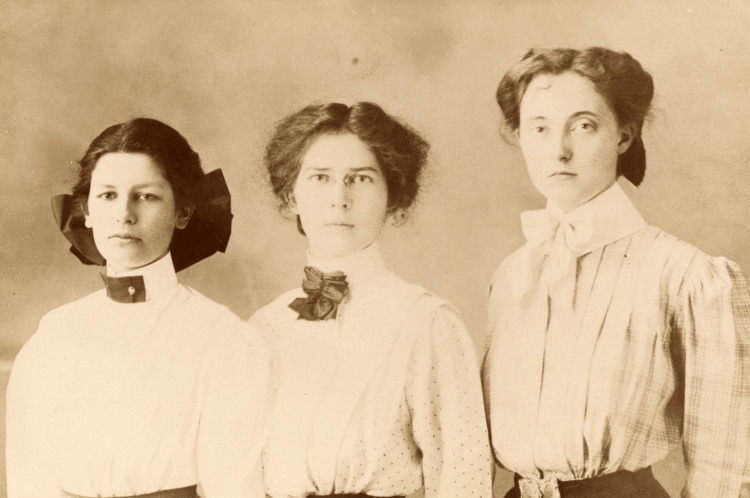
A 1909 photo of the Albany College debate team, with Lucille (Alan) Hart on the right. Used under a Creative Commons Attribution 4.0 International License (CC BY 4.0). Courtesy of Lewis & Clark College Special Collections and Archives, Dr. Alan L. Hart Collection, Portland, Oregon. Available from: https://specialcollections.lclark.edu/items/show/93 Rights information: https://creativecommons.org/licenses/by/4.0/

Hart attended Albany College and later transferred to Stanford University in 1910, where he thrived in the progressive San Francisco environment. He founded the school’s first women’s debate team (Figure 2) and reclaimed his preference for masculine dress and mannerisms. He was admitted to the University of Oregon Medical School in 1913 and graduated first in his class in 1917, earning the Saylor Medal for outstanding academic achievement [1,2,3].

Gender transition and personal challenges

While excelling academically, Hart battled deep gender dysphoria and sought help from Dr. J. Allen Gilbert, a professor of psychiatry. Initially, they pursued psychotherapy and hypnosis in hopes that Hart could conform to societal expectations of womanhood. However, these efforts failed, and Hart reportedly said the prospect of living as a woman made him physically ill. In 1917, with Gilbert’s support, Hart underwent one of the earliest known gender-affirming surgeries in the U.S. - a hysterectomy [1,4].

After surgery, Hart cut his hair, adopted male clothing, and began living openly as a man. He legally changed his name to Alan and embarked on his medical career. Dr. Gilbert later published an anonymous case study documenting the process, stating, “She made her exit as a female and started as a male with a new hold on life and ambitions worthy of her high degree of intellectuality” [5].

**Figure 3:**
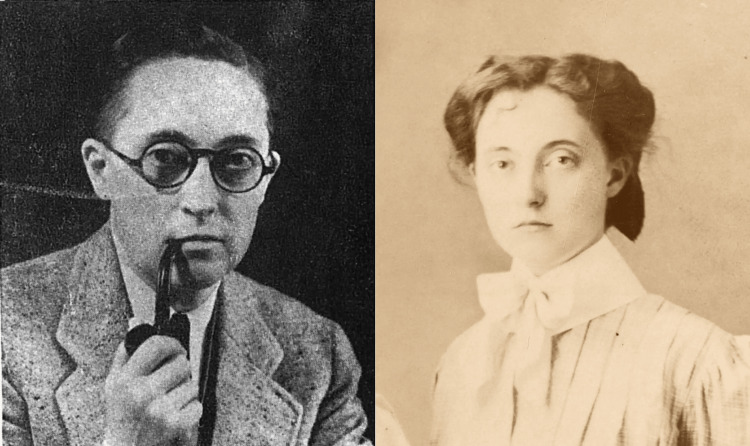
Dr. Alan L. Hart, 1942, after gender-affirming surgery (Left); Lucille Hart, 1909, before transitioning (Right). Used under a Creative Commons Attribution 4.0 International License (CC BY 4.0). Courtesy of Lewis & Clark College Special Collections and Archives, Dr. Alan L. Hart Collection, Portland, Oregon. Retrieved from: https://college.lclark.edu/live/news/43320-from-the-archives-dr-alan-hart. Rights information: https://creativecommons.org/licenses/by/4.0/

Despite this courageous transition, Hart’s early professional life was fraught with discrimination. While working as an intern at San Francisco Hospital, he was outed by a former classmate. In 1918, the San Francisco Examiner ran a sensationalist headline: “Intern Unmasked as Girl Graduate of Oregon School” [2]. Hart was forced to resign and return to Oregon. Soon after, in an interview for the Albany Daily Democrat, Hart stated, “I had to do it. For years I had been unhappy. With all the inclinations and desires of the boy I had to restrain myself to the more conventional ways of the other sex. I have been happier since I made this change than I ever have in my life, and I will continue this way as long as I live. Very few people can understand…, and I have had some of the biggest insults of my career…. I am ashamed of nothing.” [1]. Figure 3 offers a visual comparison of Hart before and after his gender transition.

Between 1918 and 1925, Hart held medical positions across several states, often relocating due to public exposure and harassment. He and his first wife, Inez Stark, divorced in 1925 due to the strain. That same year, he married Edna Ruddick, a social worker, with whom he remained until his death [1,2].

## Review

Medical contributions and public health impact

Hart developed a specialty in radiology at a time when tuberculosis (TB) was one of the leading causes of death in the U.S. Recognizing that X-rays could identify TB before symptoms appeared, he pioneered the use of chest radiography as a screening tool. This was revolutionary: before Hart, chest X-rays were primarily used only after serious symptoms had developed [3,6].

He earned a Master’s in Radiology from the University of Pennsylvania in 1928 and, later, a Master’s in Public Health from Yale in 1948. As Director of Hospitalization and Rehabilitation for the Connecticut State Tuberculosis Commission, Hart oversaw statewide screening programs, using mobile X-ray units to detect asymptomatic TB cases. These initiatives led to earlier treatment and lower transmission rates - saving countless lives [1,4,6].

**Figure 4:**
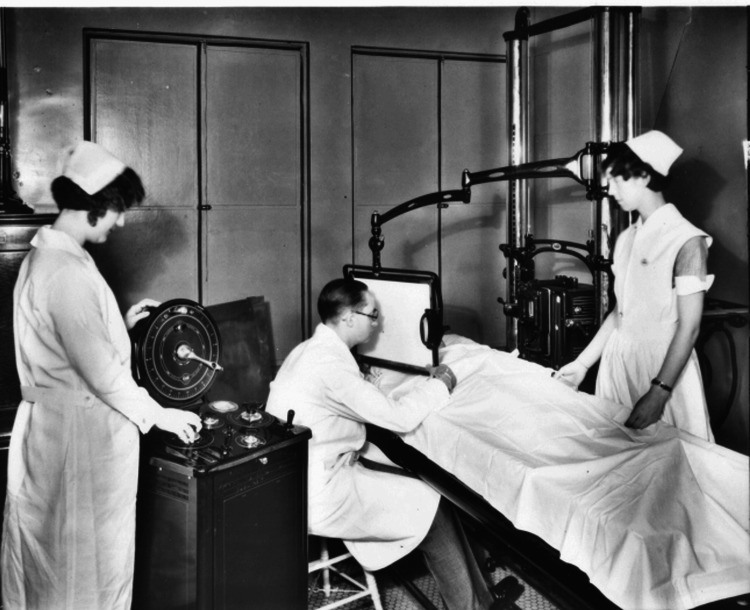
Dr. Alan Hart is depicted operating an X-ray machine at Tacoma General Hospital in the early 1930s, highlighting his contributions to medical imaging. Used with permission from Tacoma Public Library’s Northwest Room, Richards Studio Photographs (Collection 2.1.1), item 647-9. https://tpl.accesstomemory.org/647-9.

As seen in Figure 4, Hart was directly involved in operating X-ray technology at Tacoma General Hospital.

Hart’s public health approach emphasized early detection, community outreach, and preventive care. He published numerous articles and held leadership positions in professional organizations such as the American Public Health Association and the American Thoracic Society [1,4,6].

Discrimination and legacy in LGBTQIA+ history

**Figure 5:**
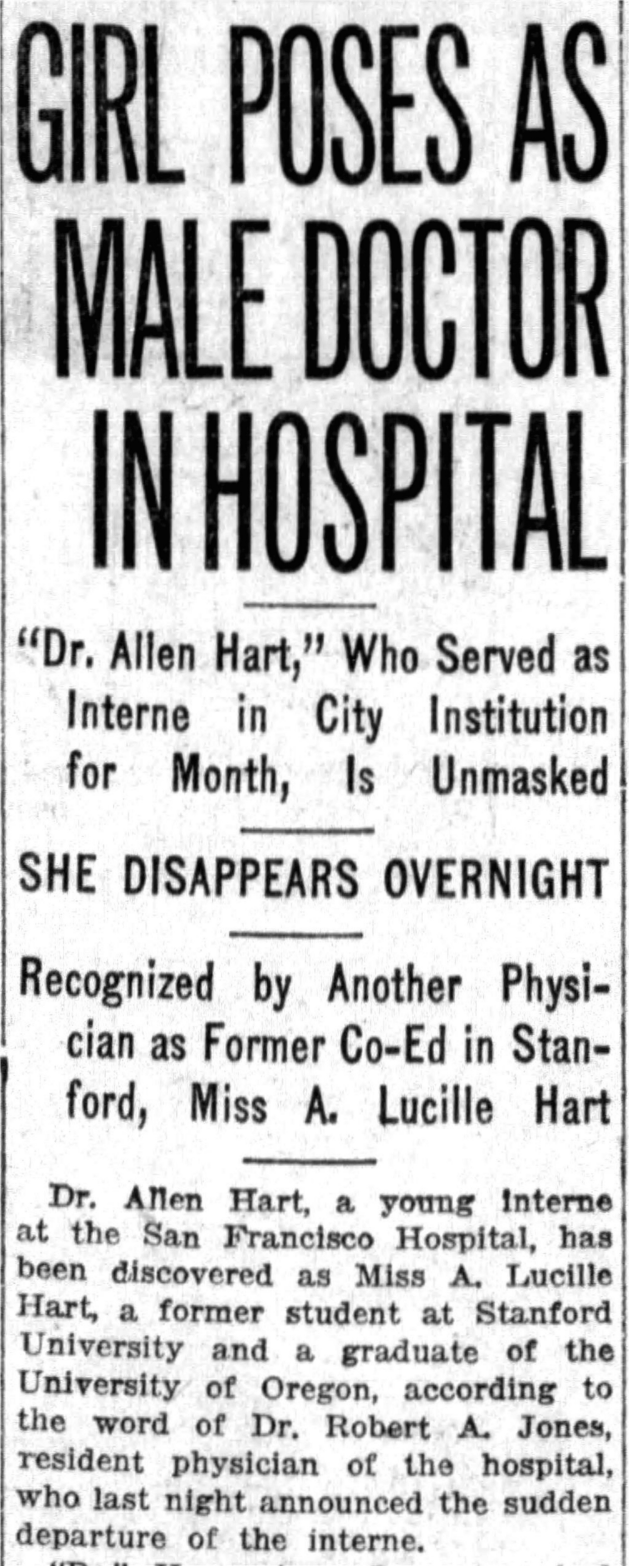
Article exposing Dr. Hart as transgender. San Francisco Examiner, February 5, 1918. Public domain image from San Francisco Examiner (February 5, 1918), courtesy of Chronicling America, Library of Congress. Rights information available at: https://www.loc.gov/collections/chronicling-america/about-this-collection/rights-and-access/.

Though Hart’s gender identity repeatedly jeopardized his employment, he remained steadfast. Newspaper exposés forced him to relocate multiple times, but he never recanted his identity (Figure 5). He was among the first to transition with medical and social affirmation, and his courageous visibility has since inspired generations of transgender individuals [2,4,7].

Hart’s life demonstrates the compounded challenges faced by lesbian, gay, bisexual, transgender, queer, intersex, asexual, and other non-binary or non-heterosexual identities (LGBTQIA+) professionals, particularly in medicine. Despite persistent stigma, he carved out a successful career and lived authentically. His posthumous inclusion in Chicago’s Legacy Walk underscores his impact as a pioneer in both medicine and LGBTQIA+ history [2,7]. According to HistoryLink, Hart’s contributions extended beyond medicine into broader public visibility for transgender professionals [8].

Literary contributions

**Figure 6:**
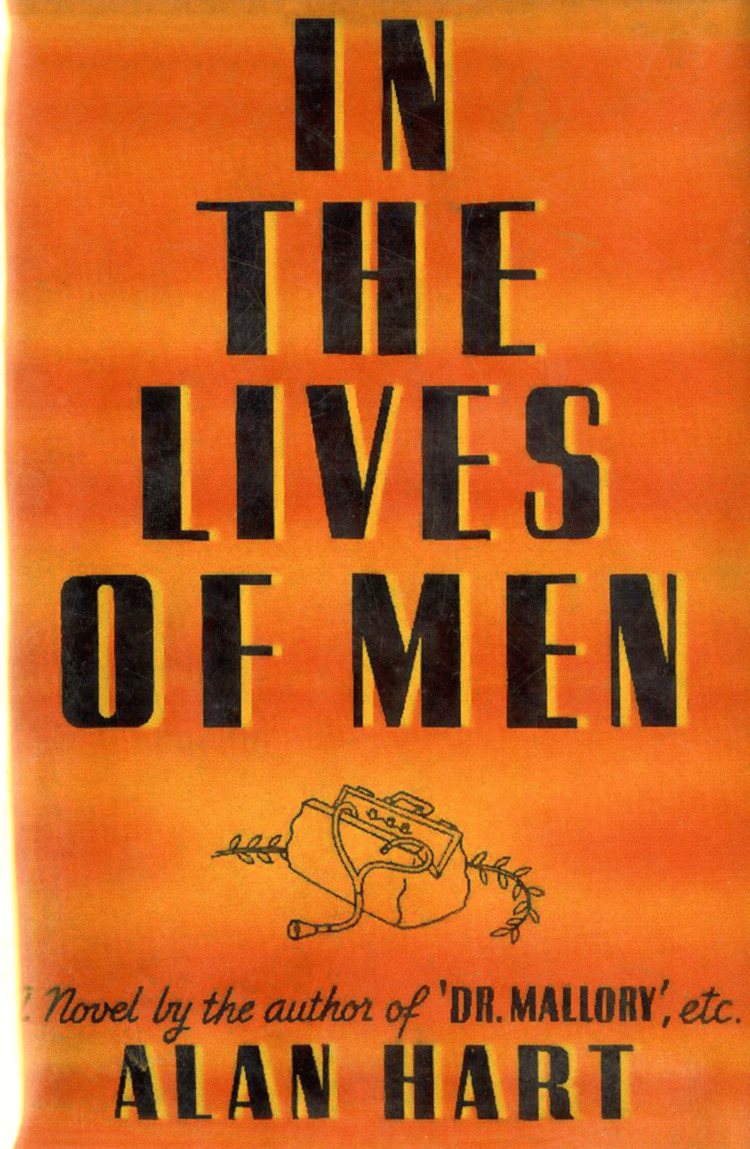
The cover of In The Lives of Men (1937) by Alan Hart reflects the themes explored in his literary work Used under a Creative Commons Attribution 4.0 International License (CC BY 4.0). Courtesy of Lewis & Clark College Special Collections and Archives, Dr. Alan L. Hart Collection, Portland, Oregon. Available from: https://specialcollections.lclark.edu/items/show/102 Rights information: https://creativecommons.org/licenses/by/4.0/

Hart was also a novelist, writing under his own name and the pen name Robert Allen Bamford Jr. His works, including Doctor Mallory and The Undaunted, feature themes of medical ethics, social injustice, and identity. One novel’s character, a radiologist who is harassed for his perceived sexual identity, mirrors Hart’s own experiences (Figure 6) [1,3,4].

His novels were critically reviewed and provided subtle commentary on the social and professional challenges of his time. Through fiction, Hart processed the realities of being a marginalized professional while advocating for ethical medicine [1,6].

## Conclusions

Dr. Alan L. Hart was a visionary physician and transgender pioneer whose work in TB detection transformed public health. His life was marked by profound courage, intellectual brilliance, and resilience in the face of discrimination. Today, Hart’s story remains an essential part of medical and LGBTQIA+ history, reminding us that authenticity and scientific excellence are not mutually exclusive but mutually reinforcing.

His legacy lives on through TB screening practices, literary contributions, and the growing visibility of transgender professionals in medicine. Alan L. Hart broke barriers that continue to resonate more than a century later.
